# Predictive performance of a multivariable difficult intubation model for obese patients

**DOI:** 10.1371/journal.pone.0203142

**Published:** 2018-08-30

**Authors:** Arunotai Siriussawakul, Patcharee Maboonyanon, Subongkot Kueprakone, Suthasinee Samankatiwat, Chulaluk Komoltri, Chayanan Thanakiattiwibun

**Affiliations:** 1 Department of Anesthesiology, Faculty of Medicine, Siriraj Hospital, Mahidol University, Bangkok, Thailand; 2 Division of Anesthesiology, Phahonpolpayuhasena Hospital, Kanchanaburi, Thailand; 3 Division of Anesthesiology, Taksin Hospital, Bangkok, Thailand; 4 Division of Anesthesiology, Ratchaburi Hospital, Ratchaburi, Thailand; 5 Division of Clinical Epidemiology, Department of Research Development, Faculty of Medicine, Siriraj Hospital, Mahidol University, Bangkok, Thailand; 6 Integrated Perioperative Geriatric Excellent Research Center, Faculty of Medicine, Siriraj Hospital, Mahidol University, Bangkok, Thailand; Indiana University, UNITED STATES

## Abstract

**Background:**

A predictive model of scores of difficult intubation (DI) may help physicians screen for airway difficulty to reduce morbidity and mortality in obese patients. The present study aimed to set up and evaluate the predictive performance of a newly developed, practical, multivariate DI model for obese patients.

**Methods:**

A prospective multi-center study was undertaken on adults with a body mass index (BMI) of 30 kg/m^2^ or more who were undergoing conventional endotracheal intubation. The BMI and 10 preoperative airway tests (namely, malformation of the teeth in the upper jaw, the modified Mallampati test [MMT], the upper lip bite test, neck mobility testing, the neck circumference [NC], the length of the neck, the interincisor gap, the hyomental distance, the thyromental distance [TM] and the sternomental distance) were examined. A DI was defined as one with an intubation difficulty scale (IDS) score ≥ 5.

**Results:**

The 1,015 patients recruited for the study had a mean BMI of 34.2 (standard deviation: 4.3 kg/m^2^). The proportions for easy intubation, slight DI and DI were 81%, 15.8% and 3.2%, respectively. Drawing on the results of a multivariate analysis, clinically meaningful variables related to obesity (namely, BMI, MMT, and the ratio of NC to TM) were used to build a predictive model for DI. Nevertheless, the best model only had a fair predictive performance. The area under the receiver operating characteristic curve (AUC) was 0.71 (95% confidence interval 0.68–0.84).

**Conclusions:**

The predictive performance of the selected model showed limited benefit for preoperative screening to predict DI among obese patients.

## Introduction

The reported incidence of difficult intubation (DI) among obese subjects varies from 1.8% to 14.3% [1–3]. These figures are much higher than the incidence reported for the general patients enrolled in the Perioperative Anesthetic Adverse Events in Thailand (PAAD THAI) Study, which was only 8:10,000, or 0.08% [4]. Obese patients experience a range of physiological alterations, including an increased oxygen consumption, a decrease in chest wall compliance and a reduction in functional residual capacity [5]. As expected in difficult situations, a long intubation time or low oxygen saturation may result in a high risk of perioperative adverse events, including death, persistent brain damage, unnecessary tracheostomy and unanticipated Intensive Care Unit admission [6].

Difficult intubation is commonly predicted using the Mallampati classification, thyromental distance, sternomental distance and interincisor gap. Nevertheless, the pooled sensitivity of each method is poor to moderate (range: 22%–62%) [7]. A combination of each test, or building risk scores, may provide high sensitivity; in other words, the model would have the ability to discriminate obese patients who have no outstanding features of problematic patients in non-difficult conditions. Some existing models, namely, the Nuguib and Arné models, have revealed a good performance in the prediction of difficult intubations [8, 9]. The initial version of the Arné model comprised variables obtained from patient histories and physical examinations. The selection of those variables may lead to multicollinearity or interaction between the variables in the model because patients’ diseases are commonly the single factor determining the airway pathology. For example, severe diabetic mellitus is related with limited joint ability or stiff joint syndrome. Acromegaly is related with macroglossia, prognathism and abnormal glottic structures, while rheumatoid arthritis is related with cervical spine abnormalities. In addition, some variables in the model were subjective, making them difficult to be interpreted by trainees or non-anesthesiologist personnel and leading to limitations in the model’s application in clinical practice. As for the Neguib model, the authors have provided the model with the highest sensitivity to date for predicting unanticipated DI. However, some variables in the final model were not related to obese patients. Therefore, the current study set out to establish and assess the predictive performance of a new, practical, multivariable DI model for patients suffering from obesity.

## Materials and methods

This prospective, observational, multi-center study involved 1 university hospital and 4 tertiary-care hospitals. It was authorized by the Siriraj Institutional Review Board, and patients gave their informed consent in writing. The enrolled patients comprised adults who were obese (defined as a BMI ≥ 30 kg/m^2^) and scheduled to undertake elective surgery requiring general anesthesia using standard endotracheal intubation. Any patient with an obvious upper airway malformation or a history of difficult or failed intubations was excluded.

In order to consider all airway assessment tests which could be used to predict a difficult intubation, a literature review was undertaken. The search terms utilized were (“difficult intubation” OR “difficult airway”) AND (“prediction” or “risk factor” OR “predictive model”) AND airway assessment AND obesity, and other such combinations. Table 1 summarizes the definitions of airway assessment which were not specific to obese patients obtained from the literature. Five anesthesiologists, each with at least 5 years’ clinical experience, developed clear definitions for each of 10 preoperative airway assessment methods (malformation of the teeth in the central part of the maxilla; modified Mallampati classification; hyomental, thyromental and sternomental distances; interincisor gap; range of motion, circumference and length of the neck; and upper lip bite test). Before the study commenced, 10 research assistants were trained in the examination modes, utilizing 5 volunteers who were obese and sets of photographs for that purpose. The instruction sessions continued until the interobserver reliabilities of the principle investigator and the 10 research assistants exceeded 0.7.

**Table 1:** Description of airway assessment tests reported for general and obese patients.

Tests	Definition
Malformation of teeth	Buck [19], protruded or missing central teeth in the upper jaw [20].
Interincisor gap	The maximal distance between the upper and lower incisors, measured while patients sit in the neutral position [21].
Upper Lip Bite test	Class I: lower incisors can bite the upper lip above the vermillion line. Class II: lower incisors can bite the upper lip below the vermillion line. Class III: lower incisors cannot bite the upper lip [22].
Modified Mallampati test	The patients sit upright with the head in the neutral position, and open their mouths as wide as possible and protrude their tongues to the maximum, and without phonation. Class I: the soft palate, fauces, uvula and pillars can be seen. Class II: the soft palate, fauces and uvula can be seen. Class III: if only the soft palate and base of the uvula can be seen. Class IV: if the soft palate is not visible [22].
Hyomental distance	The distance just above hyoid bone to the tip to the anterior-most part of the mentum in the neutral position [23].
Neck circumference	The level of the cricoid cartilage, perpendicular to the long axis of the neck [24].
Length of neck	The length from the external occipital protuberance to the vertebra prominens, as well as the circumference at the level of the cricoid cartilage anteriorly and spinous process of the sixth cervical vertebra posteriorly [19].
Neck mobility testing	Sagittal flexion: the subjects are required to make a ‘‘double chin” (suboccipital flexion) and then flex fully forward. Sagittal extension: nodding the head back and then fully extending it [25].
Thyromental distance	The straight line between the thyroid notch and the bony point of the mentum with the head fully extended, measured in the supine position with the head fully extended and the mouth closed [22, 26].
Sternomental distance	The straight distance between the upper border of the manubrium sterni and the bony point of the mentum, measured in a seated position with the head fully extended and the mouth closed [22].

We specified the following four desirable attributes of a predictive model. Firstly, the selected predictive factors should be obtained from a physical examination. In addition, the tests must be easy enough to undertake to allow assessment at a bedside or in a preoperative clinic. Moreover, only a tape measure and a ruler should be required for the assessment; no complex apparatus should be needed. Lastly, the final score should be calculated easily and be user friendly.

### Anesthetic protocol

Standard monitoring, namely, the use of a pulse oximeter, an electrocardiogram and non-invasive blood pressure, was employed before administering anesthesia. All tracheal intubations were conducted by anesthetists or anesthesiologists who had at least 2-years’ experience in a fulltime capacity and were blinded to the details of the individual patient assessments. In addition, the staff chose the laryngoscope position and the technique for intubation that they deemed would provide the best achievable visualization. The first laryngoscopy employed size 3 or 4 Macintosh laryngoscope blades. Patients were positioned with pillows supporting their heads and with their necks extended. The termination of intubation, i.e., the point when it was decided to cease standard intubation or to select alternative medical devices for airway management, was determined by the anesthesiologists in-charge. All patients were preoxygenated with 100% oxygen via a facemask for at least 3 minutes. The induction of the general anesthesia was achieved with either 1.5–2.5 mg/kg propofol or 5–7 mg/kg sodium thiopental, coupled with intubating dosages of muscle relaxants.

### Definition of difficult intubation

**Fig 1:**
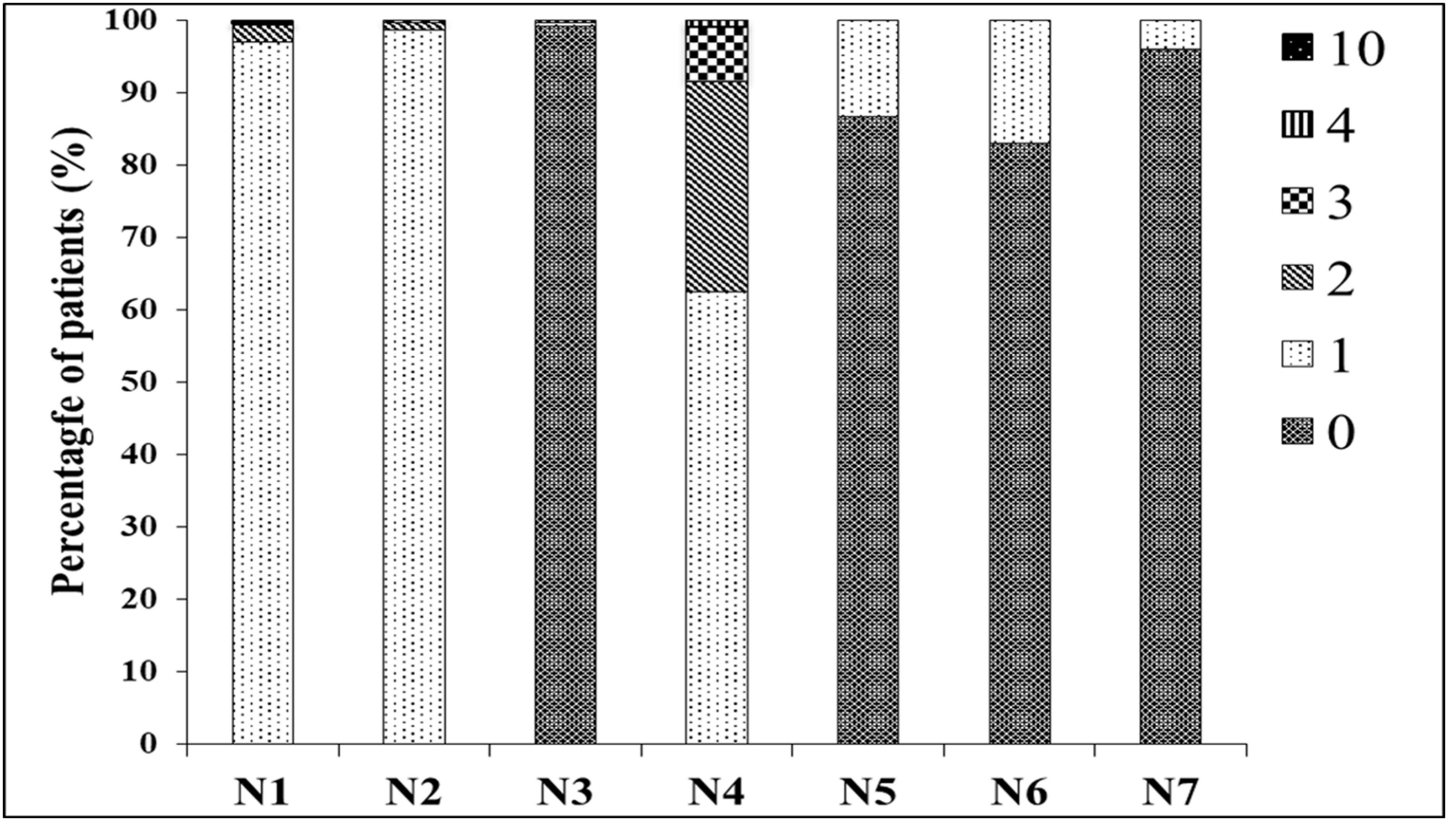
Seven components of the intubation difficulty scale (IDS). N1 represents the number of intubation attempts; N2 the number of operators; N3 the number of alternative techniques; N4 the Cormack and Lehane laryngoscopic views; N5 the lifting force applied during laryngoscopy (score 0 if considerable; score 1 if considerable); N6 the necessity to apply external laryngeal pressure to optimize glottic exposure (score 0 if no external pressure or if only the Sellick maneuver was applied; and N7 the visibility of the laryngeal aperture (score 0 if abducted vocal cord; score 1 if adducted vocal cord or invisible).

The intubation difficulty scale (IDS) was used in this study to avoid possible misunderstandings with the term “difficult intubation”. The IDS score is comprised of seven variables that have been reported as being related to difficult intubation (Fig 1). The parameter N1, which represents the number of intubation attempts, is most commonly associated with difficult intubation. The grading of the laryngeal view, described by Cormack and Lehane, is also an IDS score component (N4) [10]. This classification scheme is regarded as a standard tool for the description of views of the glottis. Researchers and clinicians use it to share their views on the degrees of intubation difficulties. The IDS score can also be used for comparisons of intubation difficulty levels in a variety of conditions, either through the summation of the scale’s 7 components or by examining specific variables.

The score has already been used for comparisons between the degree of intubation difficulty experienced by obese and non-obese patients [3]. A summation score of “0” represents an ideal, or easy, intubation. More specifically, it is one that is accomplished without noticeable physical exertion and in one attempt, is administered by a single operator, uses only one technique, and finds no impediment in the tube passage. The numerical value of the score climbs as further attempts are made; an impossible intubation is represented by the score of ∞ [11]. For our study of obese Thai patients, we defined a difficult intubation as having an IDS score ≥ 5 [12].

### Statistical analysis

The sample size estimate for our study drew upon the recommendations by statisticians for the performance of multiple logistic regression analysis [13, 14], namely, that the number of obese subjects with DI should be 5 to 10 times the (in this case, 11) risk factors in the multiple logistic model (namely, BMI; malformation of teeth; upper lip bite test; Mallampati classification; neck mobility, length and circumference; thyromental, hyomental and sternomental distances; and interincisor gap). Given that, 55–110 subjects with DI were required. As a previous incidence report demonstrated that there was some degree of DI in around 15% of cases, a sample size of around 1,000 cases was deemed adequate for developing a model [12].

SPSS Statistics for Windows, version 18.0 (SPSS Inc., Chicago, Ill., USA), and MedCalc Statistical Software, version 17.6 (MedCalc Software bvba, Ostend, Belgium) were employed for the statistical analyses (S1 File). The baseline demographic data were summarized according to the data type: continuous data by their means and standard deviations, and categorical data by the percentages of individuals falling into each category. The model was developed using data obtained solely from the derivation cohort. All variables that were known to be related with DI in obesity were considered. DI or no DI was compared with either the Chi-squared test or the independent samples t-test. Factors which had clinical meaningfulness and/or a p-value < 0.2 from the univariate analysis were then used for the multiple logistic regression model.

The regression coefficients obtained from the multivariable model were used to develop the predictive model. The model’s calibration, or its fit to the data, was subsequently assessed with the Hosmer–Lemeshow test; this was determined by the degree of agreement between the risk score probabilities that had been predicted by the model, and the probabilities that were actually observed [15]. We estimated the model’s prognostic ability to discriminate patients with, or without, a risk of difficult intubation using the receiver operating characteristic (ROC) curve; the estimated shrinkage factor was then tested for the performance of difficult intubation. The optimal cut-point of the predictive score was identified by the ROC curve’s shape, and the area under the curve (AUC) allowed for an estimation to be made of the degree of a test’s discriminative power. The AUC could have a value from 0 to 1, and it was a satisfactory indicator of the goodness of a test. For a diagnostic test to be regarded as perfect, it would have an AUC of 1.0; in comparison, the AUC of a nondiscriminating test would be 0.5 [16]. In addition, the maximum value of the Youden’s index was considered. This global measure of performance is used to assess a diagnostic procedure’s overall discriminative power and to compare it with others [17, 18]. Finally, the ROC curve was presented to demonstrate the performance of difficult intubation for the best cut-off point in terms of Youden’s index, sensitivity, specificity, positive predictive value (PPV), negative predictive value (NPV), positive likelihood ratio (LR+), negative likelihood ratio (LR-), AUC and 95% confidence interval (CI).

## Results

The study enrolled 1,015 obese patients during the period from May 1, 2013 to August 31, 2016. The data of 500 cases from a university hospital and of 515 additional cases from 4, non-university hospitals were collected. The patients’ mean age was 48.3 years, with around three-quarters being female. Their average BMI was 34.2±4.3 kg/m^2^ (range: 30–68.4 kg/m^2^), and two-thirds had at least one coexisting disease, the most frequent being hypertension, diabetes mellitus and dyslipidemia. Thirty-one also had obstructive sleep apnea. Details of other demographic data, the preoperative airway assessment tests, the surgical procedures employed and the sites of the surgical areas are at Table 2.

**Table 2:** Demographic data of 1,015 patients.

Variables	Mean±SD or number(%)
Age (years)	48.3±14.1
Gender female; male	777 (76.6); 238 (23.4)
ASA classification II; III	779 (76.7); 236 (23.3)
Department	
General surgery	472 (46.6)
Gynecology	176 (17.4)
Orthopedics	124 (12.3)
Eye, ENT	115 (11.5)
Neurology	46 (4.5)
Urology	46 (4.5)
Others	36 (3.2)
Comorbidities	
None	378 (37.2)
Hypertension	502 (49.5)
Diabetes mellitus	218 (21.5)
Obstructive sleep apnea	31 (3.1)
BMI (kg/m^2^)	34.2±4.3
Malformation of teeth	809 (79.7)
Interincisor gap (cm)	5.1±0.7
Upper lip bite test	
Class I, II, III	682 (67.2), 275 (27.1), 58 (5.7)
Modified Mallampati Test	
Class I, II	324 (31.9), 372 (36.7)
Class III, IV	214 (21.1), 105 (10.3)
Limited neck flexion	6 (0.6)
Limited neck extension	57 (5.6)
Hyomental distance (cm)	5.0±0.8
Neck circumference (cm)	39.0±3.9
Length of neck (cm)	11.3±2.1
Thyromental distance (cm)	9.7±1.6
Sternomental distance (cm)	16.4±2.0

Abbreviations: ASA, American Society of Anesthesiologists; BMI, body mass index; ENT, ear, nose and throat; SD, standard deviation

The number of easy intubations (IDS 0–1), slight DIs (IDS 2–4) and DIs (IDS ≥ 5) were 822 (81%), 161 (15.8%) and 32 (3.2%), respectively (Table 3). The distributions of the scores components are at Fig 1. Almost all successful intubations (99.2%) were done using direct laryngoscopy. This study had no incidents of failed intubations. The Cormack and Lehane laryngoscopic view distributions were 62.6% for grade I, 29% for grade II, 7.6% for grade III and 0.9% for grade IV. During the intubation period, a 1.3% incidence of brief desaturation was recorded. Oral structure injuries were reported by 2.7% of patients; a further 4.1% reported a sore throat. There were no reports of serious complications, such as death, brain damage or aspiration.

**Table 3:** Distribution of the intubation difficulty scale (IDS).

IDS	Degree of difficulty	n (%)
0	Easy	573 (56.5)
1	Easy	249 (24.5)
2	Slight difficult	77 (7.6)
3	Slight difficult	50 (4.9)
4	Slight difficult	34 (3.3)
5	Difficult	19 (1.9)
6	Difficult	7 (0.7)
7	Difficult	1 (0.1)
8	Difficult	1 (0.1)
10	Difficult	1 (0.1)
11	Difficult	1 (0.1)
12	Difficult	1 (0.1)
18	Difficult	1 (0.1)

Table 1 outlines the 11 factors associated with difficult intubation that have been identified in literature [19–26]. The incidence of DI among obese Thai patients is low. The rule of thumb is that only 3 factors should be selected to build a model (Table 4). Three difference binary logistic regression models were fitted.

1. MMT class III, IV + [(1.6 × NC/TM) − (2.5 × interincisor gap)] (estimated shrinkage factor of 0.9).
2. [(2.70 × MMT class III, IV) + (2.69 × NC/TM)] − morbidobesity (estimated shrinkage factor of 0.82).
3. [(10.94 × MMT class III, IV) + (10.89 × NC/TM)] − BMI.

**Table 4:** Possible factors related to difficult intubation; univariate analysis.

Variables	Mean±SD or number (%)		Odds ratio (95% CI)	p-value
	IDS < 5 (n = 983)	IDS ≥ 5 (n = 32)		
BMI (kg/m^2^)	34.2±4.4	33.6±3.0	0.96 (0.87–1.06)	0.41
Malformation of teeth				
No	782 (79.5)	27 (84.4)	1	
Yes	201 (20.5)	5 (15.6)	0.72 (0.27–1.89)	0.51
Upper lip bite test				
Class I	663 (67.4)	19 (59.4)	1	
Class II–III	320 (32.6)	13 (40.6)	1.42 (0.69–2.91)	0.34
Modified Mallampati Test				
Class I–II	680 (69.2)	16 (50)	1	
Class III–IV	303 (30.8)	16 (50)	2.24 (1.11–4.55)	0.02
Neck flexion				
Not limited	977 (99.4)	32 (100)	-	1.00
Limited	6 (0.6)	0	-	
Neck extension				
Not limited	928 (94.4)	30 (93.8)	1	
Limited	55 (5.6)	2 (6.3)	1.12 (0.26–4.83)	0.87
Interincisor gap	5.1±0.7	4.6±0.7	0.31 (0.18–0.53)	0.00
Hyomental distance (cm)	5.0±0.8	4.9±0.8	0.78 (0.50–1.22)	0.28
Neck circumference (cm)	39.1±3.7	39.7±4.0	1.04 (0.95–1.14)	0.36
Length of neck (cm)	11.3±2.1	11.3±1.4	0.98 (0.83–1.17)	0.86
Thyromental distance (cm)	9.8±1.6	8.8±1.5	0.66 (0.52–0.84)	0.00
Sternomental distance (cm)	16.4±2.1	15.6±1.7	0.81 (0.68–0.97)	0.02
NC:TM	4.1±0.8	4.6±0.9	2.04 (1.41–2.95)	0.00

Abbreviation: BMI, body mass index; SD, standard deviation; NC, neck circumference; TM, thyromental distance.

The last equation was selected because of the clinical meaningfulness of the factors, with a Hosmer–Lemeshow goodness-of-fit test of 0.50 and an estimated shrinkage factor of 0.83 (Table 5). Nevertheless, the predictive performance of the selected model was only fair.

**Table 5:** Selected factors related to difficult intubation; multivariable analysis.

Variables	β	Adjusted odds ratio (95% CI)	p-value
NC:TM	0.719	2.05 (1.40–3.01)	0.000
MMT class III, IV	0.722	2.06 (1.01–4.22)	0.048
BMI (kg/ m^2^)	-0.066	0.94 (0.85–1.04)	0.210
Predictivemodelofdifficultintubation=[(10.94×MMTclassIII,IV)+(10.89×NC/TM)]−BMI			

Abbreviations: β, regression coefficient; MMT, Modified Mallampati Test (score 0 if MMT class I or II, and score 1 if MMT class III or IV); NC, neck circumference; TM, thyromental distance.

**Fig 2:**
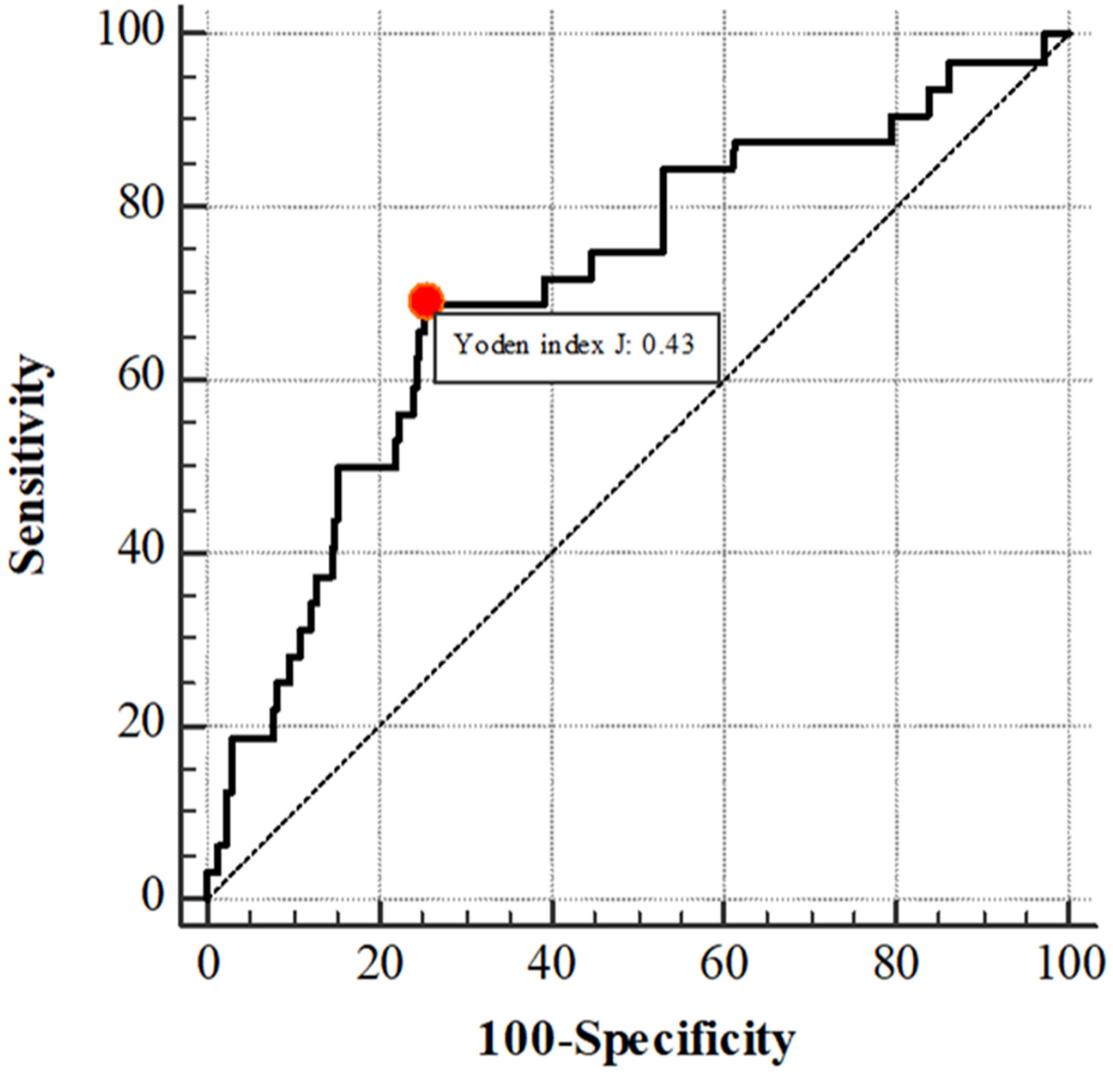
The receiver operating characteristic (ROC) curve of the predictive model of difficult intubation among obese patients.

As demonstrated at Fig 2, the AUC for the best-performing equation stood at 0.72 (95% CI 0.62–0.81). As to the cut-off point to discriminate between a high and low probability of difficult intubation, the optimal cut-off point was > 21.06. This cut-off point demonstrated the highest value of Youden’s index of 0.43; the best AUC of 0.72 (95% CI 0.62–0.81); an optimal value of sensitivity (68.75%) and specificity (74.47%); a PPV of 23.0; an NPV of 95.5; an LR+ of 2.69; and an LR- of 0.42 (Table 6).

**Table 6:** Receiver operating characteristic curve analysis.

Cut-point	Sensitivity (%) (95% CI)	Specificity (%) (95% CI)	PPV	NPV	LR+	LR-	AUC (95% CI)	Youden’s index
> 2.71	96.87 (83.8–99.9)	13.73 (11.6–16.0)	11.1	97.5	1.12	0.23	0.55 (0.46–0.64)	0.11
> 4.58	90.62 (75.0–98.0)	20.55 (18.1–23.2)	11.2	95.2	1.14	0.46	0.56 (0.46–0.65)	0.11
> 11.78	84.37 (67.2–94.7)	46.80 (43.6–50.0)	15.0	96.4	1.59	0.33	0.66 (0.57–0.74)	0.31
> 11.89	78.12 (60.0–90.7)	46.90 (43.7–50.1)	14.1	95.1	1.47	0.47	0.63 (0.53–0.72)	0.25
> 14.73	75.00 (56.6–88.5)	55.34 (52.2–58.5)	15.7	95.2	1.68	0.45	0.65 (0.56–0.74)	0.30
> 16.33	71.87 (53.3–86.3)	60.63 (57.5–63.7)	16.9	95.1	1.83	0.46	0.66 (0.57–0.75)	0.33
**> 21.06**	**68.75** **(50.0–83.9)**	**74.47** **(71.6–77.2)**	**23.0**	**95.5**	**2.69**	**0.42**	**0.72** **(0.62–0.81)**	**0.43**
> 21.27	65.62 (46.8–81.4)	75.38 (72.6–78.0)	22.9	95.2	2.67	0.46	0.70 (0.61–0.80)	0.41
> 21.29	62.50 (43.7–78.9)	75.38 (72.6–78.0)	22.0	94.8	2.54	0.50	0.69 (0.59–0.79)	0.38
> 21.36	62.50 (43.7–78.9)	75.58 (72.8–78.2)	22.1	94.8	2.56	0.50	0.69 (0.59–0.79)	0.38

Abbreviations: PPV, positive predictive value; NPV, negative predictive value; LR+, positive likelihood ratio; LR-, negative likelihood ratio; AUC, area under curve.

## Discussion

The discrimination ability of risk prediction modeling depends on the factors selected for the target population. We compared our model to two models which had been derived from studies conducted among the general population. The three equations had similar factors: the first, the Naguib model:

$$\left[\begin{array}{l} 4.9504+\left(\text{thyrosternal}\;\text{distance}\times 1.1003\right)+\left(\text{Mallampati}\;\text{score}\times \left(-2.6076\right)\right)\\ +\left(\text{thyrosternal}\;\text{distance}\times 0.9684\right)+\left(\text{neck}\;\text{circumference}\times \left(-0.3966\right)\right) \end{array}\right]$$

and the second, the new Naguib model[8]:

$$\left[\begin{array}{l} 0.2262-\left(0.4621\times \text{thyromental}\;\text{distance}\right)+\left(\left(2.5516\times \text{Mallampati}\;\text{score}\right)\right)\\ -\left(1.1461\times \text{interincisor}\;\text{gap}\right)+\left(0.0433\times \text{height}\right) \end{array}\right]$$

**Fig 3:**
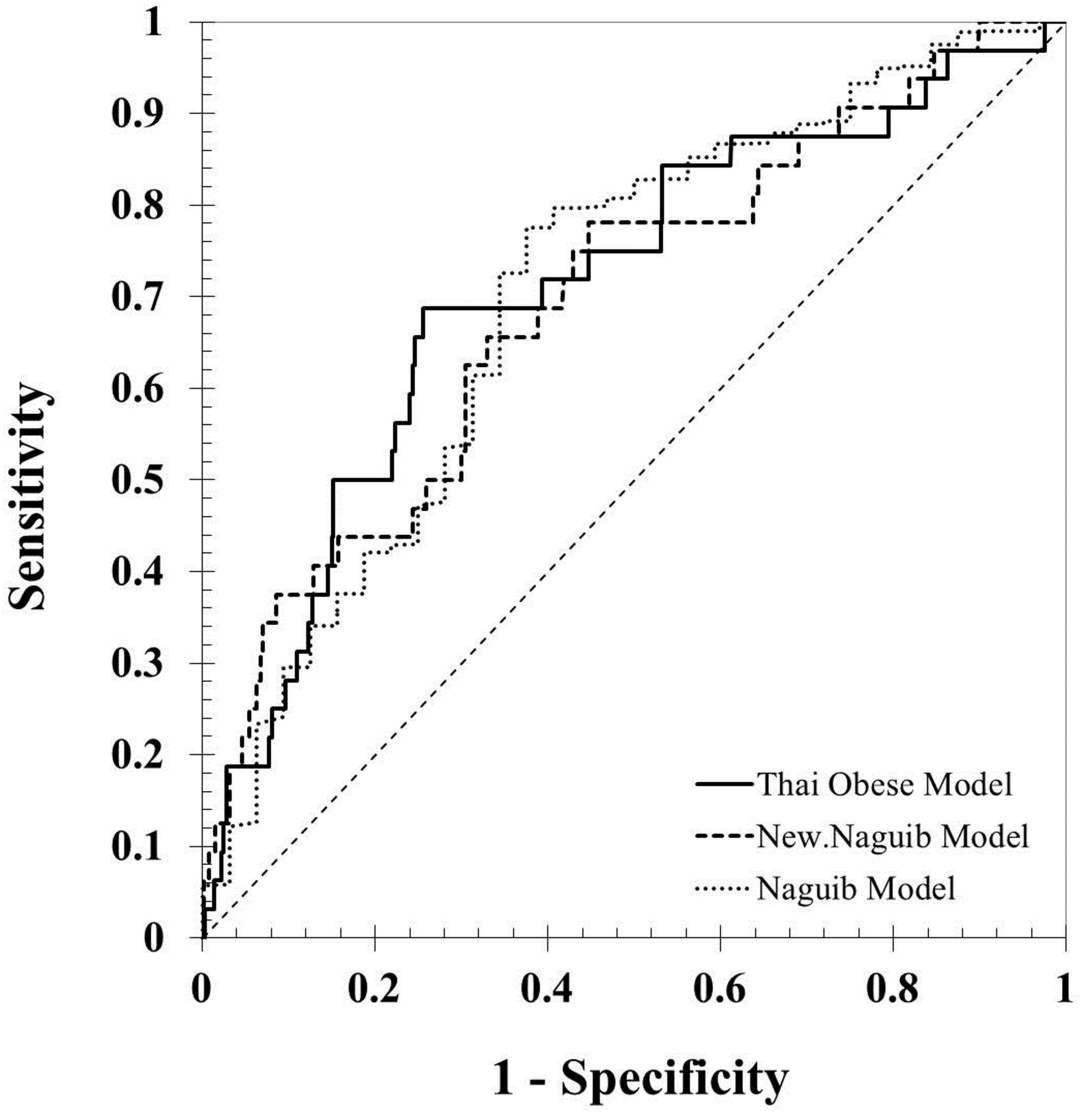
A comparison of the area under the receiver operating characteristic curve (AUC) of the three equations.

It was found that the three equations have factors associated with DI in terms of the Mallampati classification. Other possible predictors of DI in obese patients, namely, neck circumference [27], thyromental distance, BMI [3] and NC/TM [24], have been reported in other studies. However, three factors (MMT, BMI, and NC/TM) were selected for the final Thai obese model. Peduzzi et al. recommended that 10 events per predictor variable be used to prevent the major problem with the logistic model of a lack of validity. The interincisor gap was not selected to create our obese model because it is not related to obesity, even though there is a statistical significance [13]. Fig 3 shows three ROC curves representing how well the equations separate obese patients with and without DI. Overall, the accuracy of discrimination of the three presenting model equations were fair (Table 7).

**Table 7:** Predictive value of the three models tested.

Model	AUC (95% CI)	Sensitivity (%) (95% CI)	Specificity (%) (95% CI)	PPV	NPV	LR+	LR-
Thai Obese Model	0.71 (0.68–0.74)	68.75 (50.0–83.9)	74.47 (71.6–77.2)	23.0	95.5	2.69	0.42
Naguib Model	0.70 (0.67–0.73)	62.50 (43.7–78.9)	77.52 (74.8–80.1)	23.6	94.9	2.78	0.48
New Naguib Model	0.70 (0.67–0.72)	78.12 (60.0–90.7)	56.24 (52.1–58.4))	16.2	95.8	1.75	0.40

Abbreviations: AUC, area under curve; PPV, positive predictive value; NPV, negative predictive value; LR+, positive likelihood ratio; LR-, negative likelihood ratio.

Different groups of patients are associated with the selection of the factors predicting DI. With emergency ward patients, DI have been predicted using the PreDAIT model. The cut-point was ≥ 2 of the derivation set, and the validation set had an AUC of 0.68 (95% CI 0.64–0.73) and 0.63 (95% CI 0.58–0.68), with specificities of 91.5% and 87.7%, while the sensitivities were reduced to only 27.1% and 28.9%, respectively. The selection of factors was associated with emergency patients. The most parsimonious model included 5 factors: a score > 3 on the Glasgow Coma Scale (GCS); limited movement of the neck; inability to palpate the neck landmarks; trismus; and blood and/or emesis in the airway [28]. Regarding ICU patients, factors related to DI resembled factors identifiable in the operating room. The MACOCHA score draws upon the following items: MMT scores III and IV, obstructive sleep apnea syndrome, reduced cervical spine mobility, mouth opening < 3 cm, a GCS < 8, severe hypoxemia (< 80%) before intubation, and intubation by a non-anesthesiologist. The simplified scoring system had high discriminative ability, with an AUC for the validating model of 0.86 (95% CI 0.76–0.96) [29].

With regard to the characteristics of a model for screening tests, the best equations must be able to distinguish between difficult and easy intubations among patients. Consequently, a prediction model’s sensitivity is more critical than its specificity; given that, sensitivity should be weighted as being more important when deciding which model is the most appropriate. The classification by AUC for a diagnostic test, developed by Zhu et al., [30] may be summarized as excellent: 0.9 < AUC < 1.0; good: 0.8 < AUC < 0.9; worthless: 0.7 < AUC < 0.8; and not good: 0.6 < AUC < 0.5. The separation of DI among obese patients using the ROC curve showed that the AUC was 0.71, which was classified as acceptable. The estimated shrinkage factor was 0.83, which was less than 0.85 [31]. Additionally, the predictive value depends on a disease’s prevalence in the population group that is being diagnosed [32]. A good model must have sufficient prevalence, high sensitivity and high specificity, and should allow diagnosis before the patient has symptoms [32, 33].

In conclusion, the prevalence of DI among obese Thai patients was low, and the predictive performance of the selected model showed limited benefit for preoperative screening to predict DI. Further studies should discover other factors that could be added to develop an improved model for predicting the likelihood of DI. Those factors could be obtained from physical examinations as well as radiologic imaging techniques, such as ultrasonography, computerized tomography scans or magnetic resonance imaging of the neck and upper airway.

## Appendix A

To demonstrate the manner in which the predictive model of difficult intubation (DI) described in this paper is used, we present two illustrative patients assessed with the following equation:

Y=[(10.94×MMTclassIII,IV)+(10.89×NC/TM)]−BMI

Where MMT = modified Mallampati test (score 0 if MMT class I or II, and score 1 if MMT class III or IV); NC/TM = ratio of the neck circumference to the thyromental distance; BMI = body mass index (weight [kg] / height [m^2^]).

The optimal cut-point of DI was ≥ 21.06.

### Case #1

A 52-year-old man, weighing 109.5 kg and 167 cm tall, was scheduled for septoplasty. On airway examination, his interincisor gap was 4.5 cm, the MMT was class III, the neck circumference was 54.5 cm, and the thyromental distance was 9.2 cm. He had no malformation of the teeth, the upper lip bite test was grade I, and the hyomental distance was 5.7 cm.

The values for this patient can be entered in the predictive model:

$$\begin{array}{ll} \text{Y} & =\left[\left(10.94\times 1\right)+\left(10.89\times 5.92\right)\right]-39.26\\ & =36.15 \end{array}$$

As the values of the discriminant function (Y) are over 21.06, the model correctly predicted difficult intubation. Nevertheless, according to the predicted probability of the model, there was only a 9.7% likelihood that this would occur (S1 Fig).

### Case #2

A 44-year-old female, weighing 89 kg and 160 cm tall, was scheduled for a thyroidectomy. On bedside examination, her interincisor gap was 5.4 cm, the MMT class I, the neck circumference was 39.5 cm, and the thyromental distance was 9.5 cm. She had no malformation of the teeth, the upper lip bite test was grade I and the hyomental distance was 5.0 cm.

The values for this patient can be entered in the predictive model:

$$\begin{array}{ll} \text{Y} & =\left[\left(10.94\times 0\right)+\left(10.89\times 4.16\right)\right]-34.77\\ & =10.53 \end{array}$$

As the values of the discriminant function (Y) are below 21.06, the model correctly predicted an easy intubation. According to the predicted probability of the model, there was only a 1.9% chance that this was likely to occur (S1 Fig).

## Supporting information

S1 Fig Distribution of the predicted probability of difficult intubation among obese patients. (TIF)

S1 File Raw data of difficult intubation among obese patients. (XLSX)
