# Supplementary figures and images for: Predictive performance of a multivariable difficult intubation model for obese patients
Source: PLoS One. 2018 Aug 30;13(8):e0203142. doi: 10.1371/journal.pone.0203142 (PMC6117055; doi:10.1371/journal.pone.0203142)

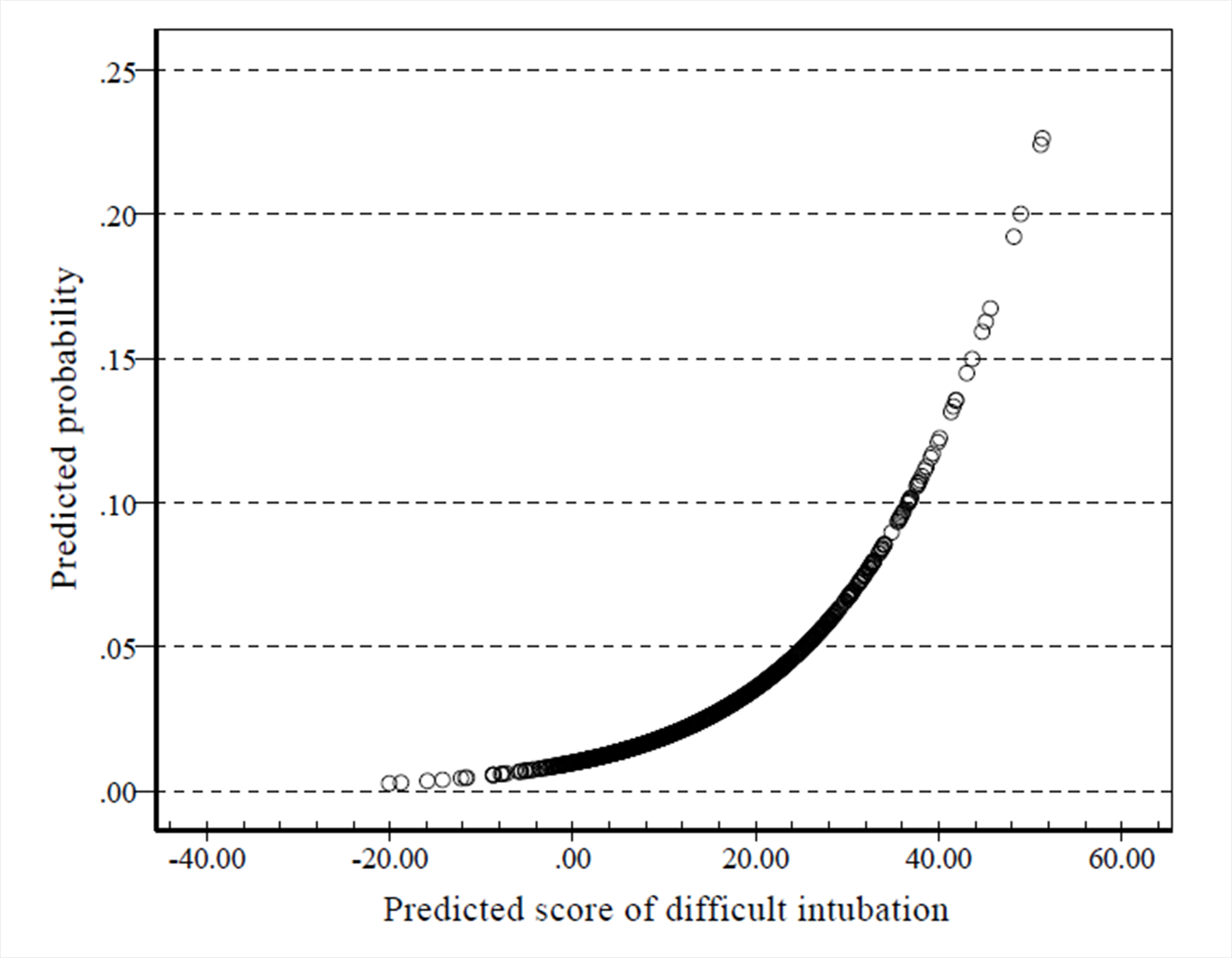

Supplement: S1 Fig — (TIF) [file pone.0203142.s001.tif]
